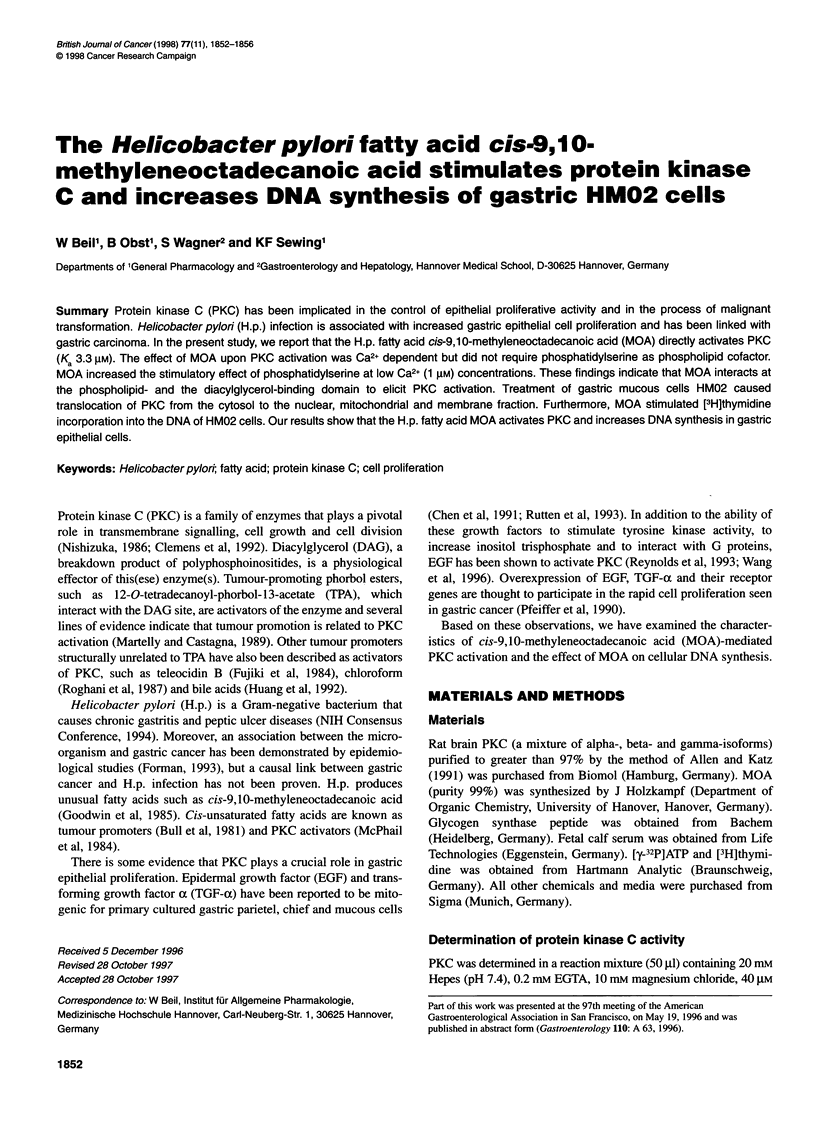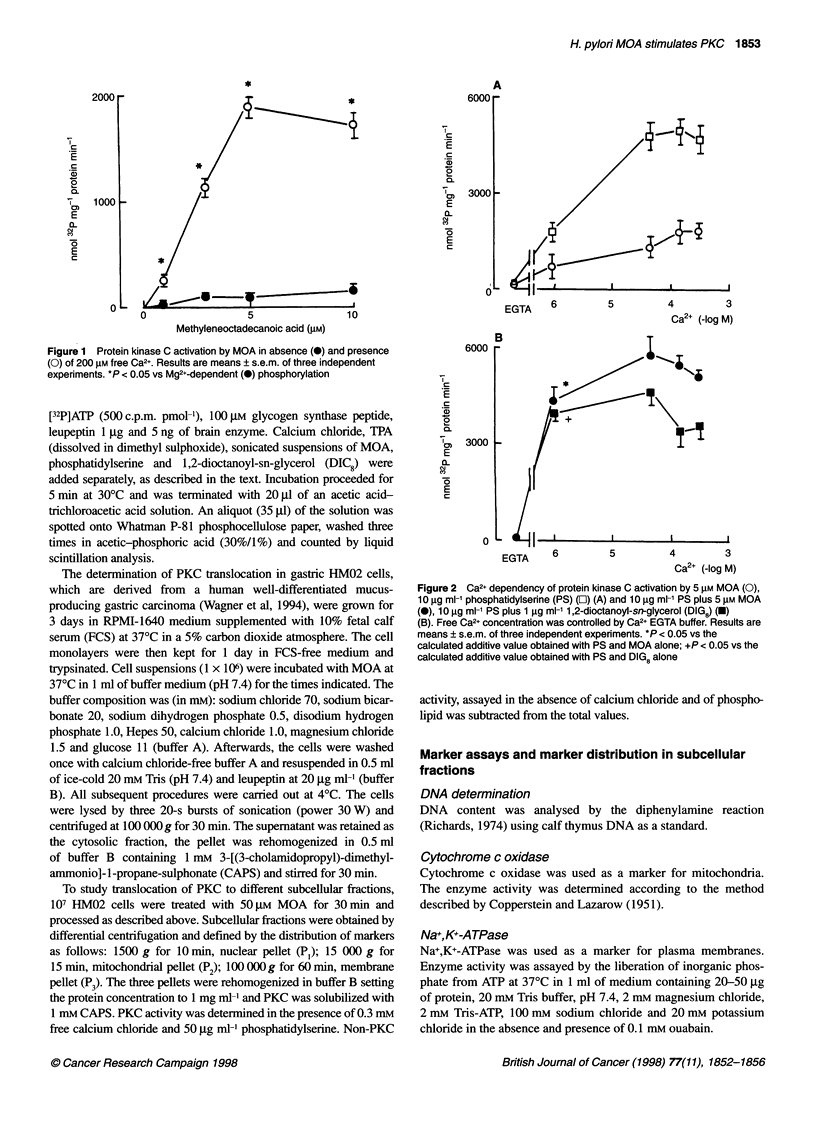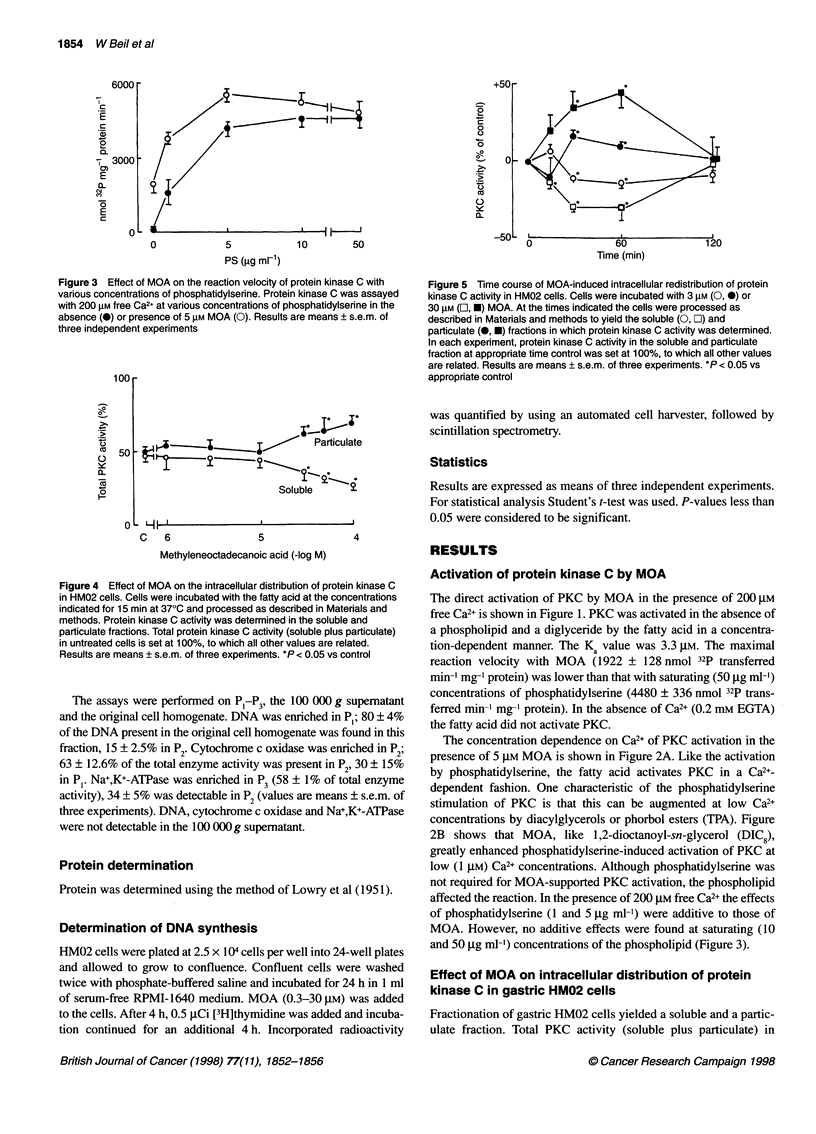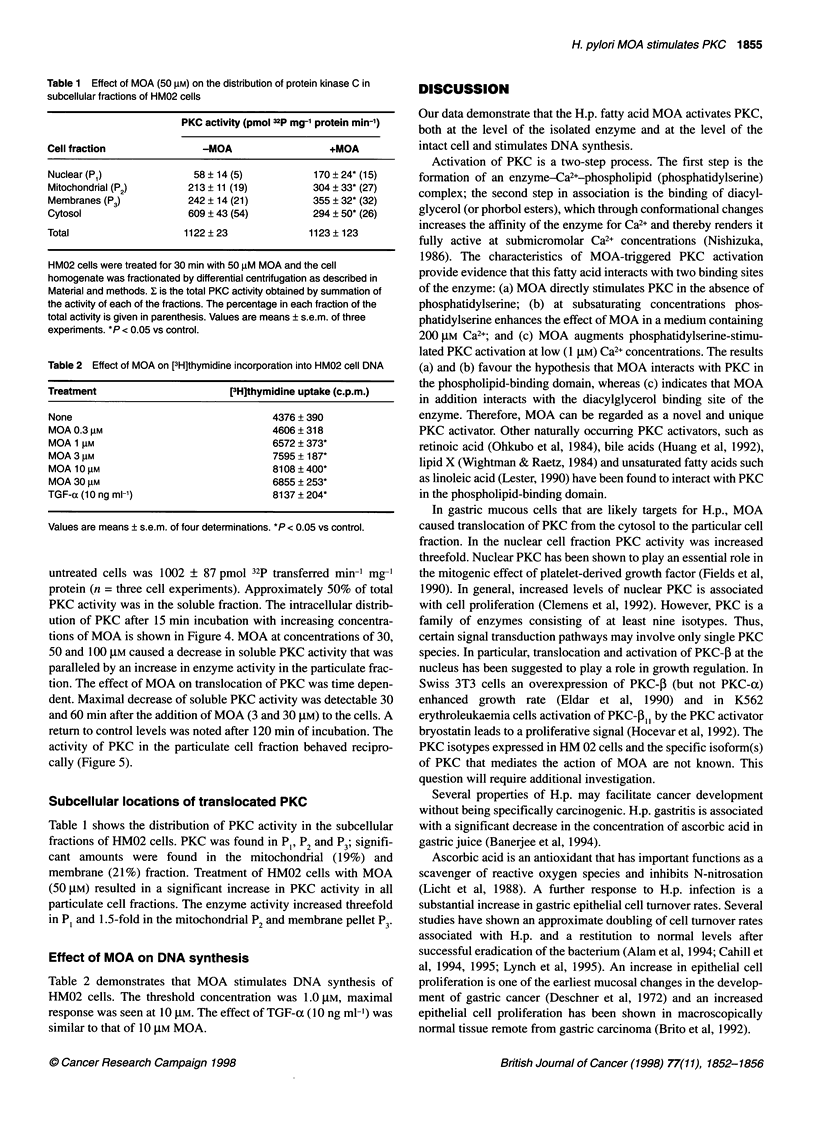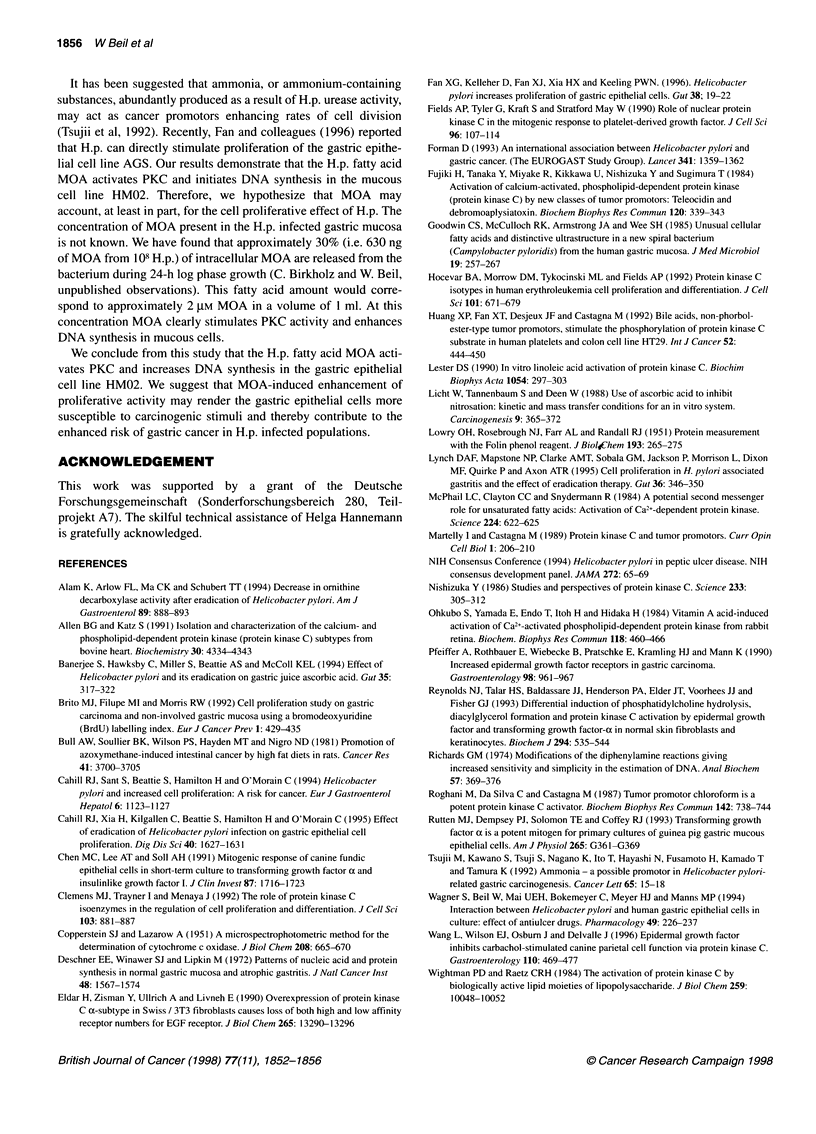# The Helicobacter pylori fatty acid cis-9,10-methyleneoctadecanoic acid stimulates protein kinase C and increases DNA synthesis of gastric HM02 cells.

**DOI:** 10.1038/bjc.1998.308

**Published:** 1998-06

**Authors:** W. Beil, B. Obst, S. Wagner, K. F. Sewing

**Affiliations:** Department of General Pharmacology, Hannover Medical School, Germany.

## Abstract

Protein kinase C (PKC) has been implicated in the control of epithelial proliferative activity and in the process of malignant transformation. Helicobacter pylori (H.p.) infection is associated with increased gastric epithelial cell proliferation and has been linked with gastric carcinoma. In the present study, we report that the H.p. fatty acid cis-9,10-methyleneoctadecanoic acid (MOA) directly activates PKC (Ka 3.3 microM). The effect of MOA upon PKC activation was Ca2+ dependent but did not require phosphatidylserine as phospholipid cofactor. MOA increased the stimulatory effect of phosphatidylserine at low Ca2+ (1 microM) concentrations. These findings indicate that MOA interacts at the phospholipid- and the diacylglycerol-binding domain to elicit PKC activation. Treatment of gastric mucous cells HM02 caused translocation of PKC from the cytosol to the nuclear, mitochondrial and membrane fraction. Furthermore, MOA stimulated [3H]thymidine incorporation into the DNA of HM02 cells. Our results show that the H.p. fatty acid MOA activates PKC and increases DNA synthesis in gastric epithelial cells.


British Journal of Cancer (1998) 77(11), 1852–1856
© 1998 Cancer Research Campaign

### The *Helicobacter pylori* fatty acid *cis*-9,10-methyleneoctadecanoic acid stimulates protein kinase C and increases DNA synthesis of gastric HM02 cells

**W Beil[1], B Obst[1], S Wagner[2] and KF Sewing[1]**

Departments of [1]General Pharmacology and [2]Gastroenterology and Hepatology, Hannover Medical School, D-30625 Hannover, Germany

**Summary** Protein kinase C (PKC) has been implicated in the control of epithelial proliferative activity and in the process of malignant transformation. *Helicobacter pylori* (H.p.) infection is associated with increased gastric epithelial cell proliferation and has been linked with gastric carcinoma. In the present study, we report that the H.p. fatty acid *cis*-9,10-methyleneoctadecanoic acid (MOA) directly activates PKC ($K_a$ 3.3 μM). The effect of MOA upon PKC activation was $Ca^{2+}$ dependent but did not require phosphatidylserine as phospholipid cofactor. MOA increased the stimulatory effect of phosphatidylserine at low $Ca^{2+}$ (1 μM) concentrations. These findings indicate that MOA interacts at the phospholipid- and the diacylglycerol-binding domain to elicit PKC activation. Treatment of gastric mucous cells HM02 caused translocation of PKC from the cytosol to the nuclear, mitochondrial and membrane fraction. Furthermore, MOA stimulated [$^3$H]thymidine incorporation into the DNA of HM02 cells. Our results show that the H.p. fatty acid MOA activates PKC and increases DNA synthesis in gastric epithelial cells.

**Keywords:** *Helicobacter pylori*; fatty acid; protein kinase C; cell proliferation

Protein kinase C (PKC) is a family of enzymes that plays a pivotal role in transmembrane signalling, cell growth and cell division (Nishizuka, 1986; Clemens et al, 1992). Diacylglycerol (DAG), a breakdown product of polyphosphoinositides, is a physiological effector of this(ese) enzyme(s). Tumour-promoting phorbol esters, such as 12-*O*-tetradecanoyl-phorbol-13-acetate (TPA), which interact with the DAG site, are activators of the enzyme and several lines of evidence indicate that tumour promotion is related to PKC activation (Martelly and Castagna, 1989). Other tumour promoters structurally unrelated to TPA have also been described as activators of PKC, such as teleocidin B (Fujiki et al, 1984), chloroform (Roghani et al, 1987) and bile acids (Huang et al, 1992).

*Helicobacter pylori* (H.p.) is a Gram-negative bacterium that causes chronic gastritis and peptic ulcer diseases (NIH Consensus Conference, 1994). Moreover, an association between the micro-organism and gastric cancer has been demonstrated by epidemiological studies (Forman, 1993), but a causal link between gastric cancer and H.p. infection has not been proven. H.p. produces unusual fatty acids such as *cis*-9,10-methyleneoctadecanoic acid (Goodwin et al, 1985). *Cis*-unsaturated fatty acids are known as tumour promoters (Bull et al, 1981) and PKC activators (McPhail et al, 1984).

There is some evidence that PKC plays a crucial role in gastric epithelial proliferation. Epidermal growth factor (EGF) and transforming growth factor α (TGF-α) have been reported to be mitogenic for primary cultured gastric parietel, chief and mucous cells (Chen et al, 1991; Rutten et al, 1993). In addition to the ability of these growth factors to stimulate tyrosine kinase activity, to increase inositol trisphosphate and to interact with G proteins, EGF has been shown to activate PKC (Reynolds et al, 1993; Wang et al, 1996). Overexpression of EGF, TGF-α and their receptor genes are thought to participate in the rapid cell proliferation seen in gastric cancer (Pfeiffer et al, 1990).

Based on these observations, we have examined the characteristics of *cis*-9,10-methyleneoctadecanoic acid (MOA)-mediated PKC activation and the effect of MOA on cellular DNA synthesis.

### MATERIALS AND METHODS

### Materials

Rat brain PKC (a mixture of alpha-, beta- and gamma-isoforms) purified to greater than 97% by the method of Allen and Katz (1991) was purchased from Biomol (Hamburg, Germany). MOA (purity 99%) was synthesized by J Holzkampf (Department of Organic Chemistry, University of Hanover, Hanover, Germany). Glycogen synthase peptide was obtained from Bachem (Heidelberg, Germany). Fetal calf serum was obtained from Life Technologies (Eggenstein, Germany). [γ-$^{32}$P]ATP and [$^3$H]thymidine was obtained from Hartmann Analytic (Braunschweig, Germany). All other chemicals and media were purchased from Sigma (Munich, Germany).

### Determination of protein kinase C activity

PKC was determined in a reaction mixture (50 μl) containing 20 mM Hepes (pH 7.4), 0.2 mM EGTA, 10 mM magnesium chloride, 40 μM

*Received 5 December 1996*
*Revised 28 October 1997*
*Accepted 28 October 1997*

*Correspondence to:* W Beil, Institut für Allgemeine Pharmakologie, Medizinische Hochschule Hannover, Carl-Neuberg-Str. 1, 30625 Hannover, Germany

Part of this work was presented at the 97th meeting of the American Gastroenterological Association in San Francisco, on May 19, 1996 and was published in abstract form (*Gastroenterology* **110**: A 63, 1996).

**Figure 1** Protein kinase C activation by MOA in absence (●) and presence (○) of 200 µM free $Ca^{2+}$. Results are means ± s.e.m. of three independent experiments. *$P < 0.05$ vs $Mg^{2+}$-dependent (●) phosphorylation

**Figure 2** $Ca^{2+}$ dependency of protein kinase C activation by 5 µM MOA (○), 10 µg $ml^{-1}$ phosphatidylserine (PS) (□) (A) and 10 µg $ml^{-1}$ PS plus 5 µM MOA (●), 10 µg $ml^{-1}$ PS plus 1 µg $ml^{-1}$ 1,2-dioctanoyl-*sn*-glycerol ($DIG_8$) (■) (B). Free $Ca^{2+}$ concentration was controlled by $Ca^{2+}$ EGTA buffer. Results are means ± s.e.m. of three independent experiments. *$P < 0.05$ vs the calculated additive value obtained with PS and MOA alone; +$P < 0.05$ vs the calculated additive value obtained with PS and $DIG_8$ alone

[$^{32}$P]ATP (500 c.p.m. $pmol^{-1}$), 100 µM glycogen synthase peptide, leupeptin 1 µg and 5 ng of brain enzyme. Calcium chloride, TPA (dissolved in dimethyl sulphoxide), sonicated suspensions of MOA, phosphatidylserine and 1,2-dioctanoyl-sn-glycerol ($DIC_8$) were added separately, as described in the text. Incubation proceeded for 5 min at 30°C and was terminated with 20 µl of an acetic acid–trichloroacetic acid solution. An aliquot (35 µl) of the solution was spotted onto Whatman P-81 phosphocellulose paper, washed three times in acetic–phosphoric acid (30%/1%) and counted by liquid scintillation analysis.

The determination of PKC translocation in gastric HM02 cells, which are derived from a human well-differentiated mucus-producing gastric carcinoma (Wagner et al, 1994), were grown for 3 days in RPMI-1640 medium supplemented with 10% fetal calf serum (FCS) at 37°C in a 5% carbon dioxide atmosphere. The cell monolayers were then kept for 1 day in FCS-free medium and trypsinated. Cell suspensions ($1 \times 10^6$) were incubated with MOA at 37°C in 1 ml of buffer medium (pH 7.4) for the times indicated. The buffer composition was (in mM): sodium chloride 70, sodium bicarbonate 20, sodium dihydrogen phosphate 0.5, disodium hydrogen phosphate 1.0, Hepes 50, calcium chloride 1.0, magnesium chloride 1.5 and glucose 11 (buffer A). Afterwards, the cells were washed once with calcium chloride-free buffer A and resuspended in 0.5 ml of ice-cold 20 mM Tris (pH 7.4) and leupeptin at 20 µg $ml^{-1}$ (buffer B). All subsequent procedures were carried out at 4°C. The cells were lysed by three 20-s bursts of sonication (power 30 W) and centrifuged at 100 000 *g* for 30 min. The supernatant was retained as the cytosolic fraction, the pellet was rehomogenized in 0.5 ml of buffer B containing 1 mM 3-[(3-cholamidopropyl)-dimethylammonio]-1-propane-sulphonate (CAPS) and stirred for 30 min.

To study translocation of PKC to different subcellular fractions, $10^7$ HM02 cells were treated with 50 µM MOA for 30 min and processed as described above. Subcellular fractions were obtained by differential centrifugation and defined by the distribution of markers as follows: 1500 *g* for 10 min, nuclear pellet ($P_1$); 15 000 *g* for 15 min, mitochondrial pellet ($P_2$); 100 000 *g* for 60 min, membrane pellet ($P_3$). The three pellets were rehomogenized in buffer B setting the protein concentration to 1 mg $ml^{-1}$ and PKC was solubilized with 1 mM CAPS. PKC activity was determined in the presence of 0.3 mM free calcium chloride and 50 µg $ml^{-1}$ phosphatidylserine. Non-PKC activity, assayed in the absence of calcium chloride and of phospholipid was subtracted from the total values.

### Marker assays and marker distribution in subcellular fractions

### *DNA determination*

DNA content was analysed by the diphenylamine reaction (Richards, 1974) using calf thymus DNA as a standard.

### *Cytochrome c oxidase*

Cytochrome c oxidase was used as a marker for mitochondria. The enzyme activity was determined according to the method described by Copperstein and Lazarow (1951).

### *$Na^+,K^+$-ATPase*

$Na^+,K^+$-ATPase was used as a marker for plasma membranes. Enzyme activity was assayed by the liberation of inorganic phosphate from ATP at 37°C in 1 ml of medium containing 20–50 µg of protein, 20 mM Tris buffer, pH 7.4, 2 mM magnesium chloride, 2 mM Tris-ATP, 100 mM sodium chloride and 20 mM potassium chloride in the absence and presence of 0.1 mM ouabain.

© Cancer Research Campaign 1998

**Figure 3** Effect of MOA on the reaction velocity of protein kinase C with various concentrations of phosphatidylserine. Protein kinase C was assayed with 200 μM free $Ca^{2+}$ at various concentrations of phosphatidylserine in the absence (●) or presence of 5 μM MOA (○). Results are means ± s.e.m. of three independent experiments

**Figure 4** Effect of MOA on the intracellular distribution of protein kinase C in HM02 cells. Cells were incubated with the fatty acid at the concentrations indicated for 15 min at 37°C and processed as described in Materials and methods. Protein kinase C activity was determined in the soluble and particulate fractions. Total protein kinase C activity (soluble plus particulate) in untreated cells is set at 100%, to which all other values are related. Results are means ± s.e.m. of three experiments. *$P < 0.05$ vs control

The assays were performed on $P_1$–$P_3$, the 100 000 *g* supernatant and the original cell homogenate. DNA was enriched in $P_1$; 80 ± 4% of the DNA present in the original cell homogenate was found in this fraction, 15 ± 2.5% in $P_2$. Cytochrome c oxidase was enriched in $P_2$; 63 ± 12.6% of the total enzyme activity was present in $P_2$, 30 ± 15% in $P_1$. $Na^+$,$K^+$-ATPase was enriched in $P_3$ (58 ± 1% of total enzyme activity), 34 ± 5% was detectable in $P_2$ (values are means ± s.e.m. of three experiments). DNA, cytochrome c oxidase and $Na^+$,$K^+$-ATPase were not detectable in the 100 000 *g* supernatant.

### Protein determination

Protein was determined using the method of Lowry et al (1951).

### Determination of DNA synthesis

HM02 cells were plated at 2.5 × $10^4$ cells per well into 24-well plates and allowed to grow to confluence. Confluent cells were washed twice with phosphate-buffered saline and incubated for 24 h in 1 ml of serum-free RPMI-1640 medium. MOA (0.3–30 μM) was added to the cells. After 4 h, 0.5 μCi [$^3$H]thymidine was added and incubation continued for an additional 4 h. Incorporated radioactivity

**Figure 5** Time course of MOA-induced intracellular redistribution of protein kinase C activity in HM02 cells. Cells were incubated with 3 μM (○, ●) or 30 μM (□, ■) MOA. At the times indicated the cells were processed as described in Materials and methods to yield the soluble (○, □) and particulate (●, ■) fractions in which protein kinase C activity was determined. In each experiment, protein kinase C activity in the soluble and particulate fraction at appropriate time control was set at 100%, to which all other values are related. Results are means ± s.e.m. of three experiments. *$P < 0.05$ vs appropriate control was quantified by using an automated cell harvester, followed by scintillation spectrometry.

### Statistics

Results are expressed as means of three independent experiments. For statistical analysis Student's *t*-test was used. *P*-values less than 0.05 were considered to be significant.

### RESULTS

### Activation of protein kinase C by MOA

The direct activation of PKC by MOA in the presence of 200 μM free $Ca^{2+}$ is shown in Figure 1. PKC was activated in the absence of a phospholipid and a diglyceride by the fatty acid in a concentration-dependent manner. The $K_a$ value was 3.3 μM. The maximal reaction velocity with MOA (1922 ± 128 nmol $^{32}$P transferred min$^{-1}$ mg$^{-1}$ protein) was lower than that with saturating (50 μg ml$^{-1}$) concentrations of phosphatidylserine (4480 ± 336 nmol $^{32}$P transferred min$^{-1}$ mg$^{-1}$ protein). In the absence of $Ca^{2+}$ (0.2 mM EGTA) the fatty acid did not activate PKC.

The concentration dependence on $Ca^{2+}$ of PKC activation in the presence of 5 μM MOA is shown in Figure 2A. Like the activation by phosphatidylserine, the fatty acid activates PKC in a $Ca^{2+}$-dependent fashion. One characteristic of the phosphatidylserine stimulation of PKC is that this can be augmented at low $Ca^{2+}$ concentrations by diacylglycerols or phorbol esters (TPA). Figure 2B shows that MOA, like 1,2-dioctanoyl-*sn*-glycerol ($DIC_8$), greatly enhanced phosphatidylserine-induced activation of PKC at low (1 μM) $Ca^{2+}$ concentrations. Although phosphatidylserine was not required for MOA-supported PKC activation, the phospholipid affected the reaction. In the presence of 200 μM free $Ca^{2+}$ the effects of phosphatidylserine (1 and 5 μg ml$^{-1}$) were additive to those of MOA. However, no additive effects were found at saturating (10 and 50 μg ml$^{-1}$) concentrations of the phospholipid (Figure 3).

### Effect of MOA on intracellular distribution of protein kinase C in gastric HM02 cells

Fractionation of gastric HM02 cells yielded a soluble and a particulate fraction. Total PKC activity (soluble plus particulate) in

© Cancer Research Campaign 1998

**Table 1** Effect of MOA (50 μM) on the distribution of protein kinase C in subcellular fractions of HM02 cells

| Cell fraction | PKC activity (pmol $^{32}P$ $mg^{-1}$ protein $min^{-1}$) | |
|---|---|---|
| | –MOA | +MOA |
| Nuclear ($P_1$) | 58 ± 14 (5) | 170 ± 24* (15) |
| Mitochondrial ($P_2$) | 213 ± 11 (19) | 304 ± 33* (27) |
| Membranes ($P_3$) | 242 ± 14 (21) | 355 ± 32* (32) |
| Cytosol | 609 ± 43 (54) | 294 ± 50* (26) |
| Total | 1122 ± 23 | 1123 ± 123 |

HM02 cells were treated for 30 min with 50 μM MOA and the cell homogenate was fractionated by differential centrifugation as described in Material and methods. Σ is the total PKC activity obtained by summation of the activity of each of the fractions. The percentage in each fraction of the total activity is given in parenthesis. Values are means ± s.e.m. of three experiments. *$P < 0.05$ vs control.

**Table 2** Effect of MOA on [$^3$H]thymidine incorporation into HM02 cell DNA

| Treatment | [$^3$H]thymidine uptake (c.p.m.) |
|---|---|
| None | 4376 ± 390 |
| MOA 0.3 μM | 4606 ± 318 |
| MOA 1 μM | 6572 ± 373* |
| MOA 3 μM | 7595 ± 187* |
| MOA 10 μM | 8108 ± 400* |
| MOA 30 μM | 6855 ± 253* |
| TGF-α (10 ng $ml^{-1}$) | 8137 ± 204* |

Values are means ± s.e.m. of four determinations. *$P < 0.05$ vs control.

untreated cells was 1002 ± 87 pmol $^{32}P$ transferred $min^{-1}$ $mg^{-1}$ protein (*n* = three cell experiments). Approximately 50% of total PKC activity was in the soluble fraction. The intracellular distribution of PKC after 15 min incubation with increasing concentrations of MOA is shown in Figure 4. MOA at concentrations of 30, 50 and 100 μM caused a decrease in soluble PKC activity that was paralleled by an increase in enzyme activity in the particulate fraction. The effect of MOA on translocation of PKC was time dependent. Maximal decrease of soluble PKC activity was detectable 30 and 60 min after the addition of MOA (3 and 30 μM) to the cells. A return to control levels was noted after 120 min of incubation. The activity of PKC in the particulate cell fraction behaved reciprocally (Figure 5).

### Subcellular locations of translocated PKC

Table 1 shows the distribution of PKC activity in the subcellular fractions of HM02 cells. PKC was found in $P_1$, $P_2$ and $P_3$; significant amounts were found in the mitochondrial (19%) and membrane (21%) fraction. Treatment of HM02 cells with MOA (50 μM) resulted in a significant increase in PKC activity in all particulate cell fractions. The enzyme activity increased threefold in $P_1$ and 1.5-fold in the mitochondrial $P_2$ and membrane pellet $P_3$.

### Effect of MOA on DNA synthesis

Table 2 demonstrates that MOA stimulates DNA synthesis of HM02 cells. The threshold concentration was 1.0 μM, maximal response was seen at 10 μM. The effect of TGF-α (10 ng $ml^{-1}$) was similar to that of 10 μM MOA.

### DISCUSSION

Our data demonstrate that the H.p. fatty acid MOA activates PKC, both at the level of the isolated enzyme and at the level of the intact cell and stimulates DNA synthesis.

Activation of PKC is a two-step process. The first step is the formation of an enzyme–$Ca^{2+}$–phospholipid (phosphatidylserine) complex; the second step in association is the binding of diacylglycerol (or phorbol esters), which through conformational changes increases the affinity of the enzyme for $Ca^{2+}$ and thereby renders it fully active at submicromolar $Ca^{2+}$ concentrations (Nishizuka, 1986). The characteristics of MOA-triggered PKC activation provide evidence that this fatty acid interacts with two binding sites of the enzyme: (a) MOA directly stimulates PKC in the absence of phosphatidylserine; (b) at subsaturating concentrations phosphatidylserine enhances the effect of MOA in a medium containing 200 μM $Ca^{2+}$; and (c) MOA augments phosphatidylserine-stimulated PKC activation at low (1 μM) $Ca^{2+}$ concentrations. The results (a) and (b) favour the hypothesis that MOA interacts with PKC in the phospholipid-binding domain, whereas (c) indicates that MOA in addition interacts with the diacylglycerol binding site of the enzyme. Therefore, MOA can be regarded as a novel and unique PKC activator. Other naturally occurring PKC activators, such as retinoic acid (Ohkubo et al, 1984), bile acids (Huang et al, 1992), lipid X (Wightman & Raetz, 1984) and unsaturated fatty acids such as linoleic acid (Lester, 1990) have been found to interact with PKC in the phospholipid-binding domain.

In gastric mucous cells that are likely targets for H.p., MOA caused translocation of PKC from the cytosol to the particular cell fraction. In the nuclear cell fraction PKC activity was increased threefold. Nuclear PKC has been shown to play an essential role in the mitogenic effect of platelet-derived growth factor (Fields et al, 1990). In general, increased levels of nuclear PKC is associated with cell proliferation (Clemens et al, 1992). However, PKC is a family of enzymes consisting of at least nine isotypes. Thus, certain signal transduction pathways may involve only single PKC species. In particular, translocation and activation of PKC-β at the nucleus has been suggested to play a role in growth regulation. In Swiss 3T3 cells an overexpression of PKC-β (but not PKC-α) enhanced growth rate (Eldar et al, 1990) and in K562 erythroleukaemia cells activation of PKC-$\beta_{II}$ by the PKC activator bryostatin leads to a proliferative signal (Hocevar et al, 1992). The PKC isotypes expressed in HM 02 cells and the specific isoform(s) of PKC that mediates the action of MOA are not known. This question will require additional investigation.

Several properties of H.p. may facilitate cancer development without being specifically carcinogenic. H.p. gastritis is associated with a significant decrease in the concentration of ascorbic acid in gastric juice (Banerjee et al, 1994).

Ascorbic acid is an antioxidant that has important functions as a scavenger of reactive oxygen species and inhibits N-nitrosation (Licht et al, 1988). A further response to H.p. infection is a substantial increase in gastric epithelial cell turnover rates. Several studies have shown an approximate doubling of cell turnover rates associated with H.p. and a restitution to normal levels after successful eradication of the bacterium (Alam et al, 1994; Cahill et al, 1994, 1995; Lynch et al, 1995). An increase in epithelial cell proliferation is one of the earliest mucosal changes in the development of gastric cancer (Deschner et al, 1972) and an increased epithelial cell proliferation has been shown in macroscopically normal tissue remote from gastric carcinoma (Brito et al, 1992).

© Cancer Research Campaign 1998

It has been suggested that ammonia, or ammonium-containing substances, abundantly produced as a result of H.p. urease activity, may act as cancer promotors enhancing rates of cell division (Tsujii et al, 1992). Recently, Fan and colleagues (1996) reported that H.p. can directly stimulate proliferation of the gastric epithelial cell line AGS. Our results demonstrate that the H.p. fatty acid MOA activates PKC and initiates DNA synthesis in the mucous cell line HM02. Therefore, we hypothesize that MOA may account, at least in part, for the cell proliferative effect of H.p. The concentration of MOA present in the H.p. infected gastric mucosa is not known. We have found that approximately 30% (i.e. 630 ng of MOA from $10^8$ H.p.) of intracellular MOA are released from the bacterium during 24-h log phase growth (C. Birkholz and W. Beil, unpublished observations). This fatty acid amount would correspond to approximately 2 $\mu$M MOA in a volume of 1 ml. At this concentration MOA clearly stimulates PKC activity and enhances DNA synthesis in mucous cells.

We conclude from this study that the H.p. fatty acid MOA activates PKC and increases DNA synthesis in the gastric epithelial cell line HM02. We suggest that MOA-induced enhancement of proliferative activity may render the gastric epithelial cells more susceptible to carcinogenic stimuli and thereby contribute to the enhanced risk of gastric cancer in H.p. infected populations.

### ACKNOWLEDGEMENT

This work was supported by a grant of the Deutsche Forschungsgemeinschaft (Sonderforschungsbereich 280, Teilprojekt A7). The skilful technical assistance of Helga Hannemann is gratefully acknowledged.

### REFERENCES

Alam K, Arlow FL, Ma CK and Schubert TT (1994) Decrease in ornithine decarboxylase activity after eradication of *Helicobacter pylori*. *Am J Gastroenterol* **89**: 888–893

Allen BG and Katz S (1991) Isolation and characterization of the calcium- and phospholipid-dependent protein kinase (protein kinase C) subtypes from bovine heart. *Biochemistry* **30**: 4334–4343

Banerjee S, Hawksby C, Miller S, Beattie AS and McColl KEL (1994) Effect of *Helicobacter pylori* and its eradication on gastric juice ascorbic acid. *Gut* **35**: 317–322

Brito MJ, Filupe MI and Morris RW (1992) Cell proliferation study on gastric carcinoma and non-involved gastric mucosa using a bromodeoxyuridine (BrdU) labelling index. *Eur J Cancer Prev* **1**: 429–435

Bull AW, Soullier BK, Wilson PS, Hayden MT and Nigro ND (1981) Promotion of azoxymethane-induced intestinal cancer by high fat diets in rats. *Cancer Res* **41**: 3700–3705

Cahill RJ, Sant S, Beattie S, Hamilton H and O'Morain C (1994) *Helicobacter pylori* and increased cell proliferation: A risk for cancer. *Eur J Gastroenterol Hepatol* **6**: 1123–1127

Cahill RJ, Xia H, Kilgallen C, Beattie S, Hamilton H and O'Morain C (1995) Effect of eradication of *Helicobacter pylori* infection on gastric epithelial cell proliferation. *Dig Dis Sci* **40**: 1627–1631

Chen MC, Lee AT and Soll AH (1991) Mitogenic response of canine fundic epithelial cells in short-term culture to transforming growth factor α and insulinlike growth factor I. *J Clin Invest* **87**: 1716–1723

Clemens MJ, Trayner I and Menaya J (1992) The role of protein kinase C isoenzymes in the regulation of cell proliferation and differentiation. *J Cell Sci* **103**: 881–887

Copperstein SJ and Lazarow A (1951) A microspectrophotometric method for the determination of cytochrome c oxidase. *J Biol Chem* **208**: 665–670

Deschner EE, Winawer SJ and Lipkin M (1972) Patterns of nucleic acid and protein synthesis in normal gastric mucosa and atrophic gastritis. *J Natl Cancer Inst* **48**: 1567–1574

Eldar H, Zisman Y, Ullrich A and Livneh E (1990) Overexpression of protein kinase C α-subtype in Swiss / 3T3 fibroblasts causes loss of both high and low affinity receptor numbers for EGF receptor. *J Biol Chem* **265**: 13290–13296

Fan XG, Kelleher D, Fan XJ, Xia HX and Keeling PWN. (1996). *Helicobacter pylori* increases proliferation of gastric epithelial cells. *Gut* **38**; 19–22

Fields AP, Tyler G, Kraft S and Stratford May W (1990) Role of nuclear protein kinase C in the mitogenic response to platelet-derived growth factor. *J Cell Sci* **96**: 107–114

Forman D (1993) An international association between *Helicobacter pylori* and gastric cancer. (The EUROGAST Study Group). *Lancet* **341**: 1359–1362

Fujiki H, Tanaka Y, Miyake R, Kikkawa U, Nishizuka Y and Sugimura T (1984) Activation of calcium-activated, phospholipid-dependent protein kinase (protein kinase C) by new classes of tumor promotors: Teleocidin and debromoaplysiatoxin. *Biochem Biophys Res Commun* **120**: 339–343

Goodwin CS, McCulloch RK, Armstrong JA and Wee SH (1985) Unusual cellular fatty acids and distinctive ultrastructure in a new spiral bacterium (*Campylobacter pyloridis*) from the human gastric mucosa. *J Med Microbiol* **19**: 257–267

Hocevar BA, Morrow DM, Tykocinski ML and Fields AP (1992) Protein kinase C isotypes in human erythroleukemia cell proliferation and differentiation. *J Cell Sci* **101**: 671–679

Huang XP, Fan XT, Desjeux JF and Castagna M (1992) Bile acids, non-phorbol-ester-type tumor promotors, stimulate the phosphorylation of protein kinase C substrate in human platelets and colon cell line HT29. *Int J Cancer* **52**: 444–450

Lester DS (1990) In vitro linoleic acid activation of protein kinase C. *Biochim Biophys Acta* **1054**: 297–303

Licht W, Tannenbaum S and Deen W (1988) Use of ascorbic acid to inhibit nitrosation: kinetic and mass transfer conditions for an in vitro system. *Carcinogenesis* **9**: 365–372

Lowry OH, Rosebrough NJ, Farr AL and Randall RJ (1951) Protein measurement with the Folin phenol reagent. *J Biol Chem* **193**: 265–275

Lynch DAF, Mapstone NP, Clarke AMT, Sobala GM, Jackson P, Morrison L, Dixon MF, Quirke P and Axon ATR (1995) Cell proliferation in *H. pylori* associated gastritis and the effect of eradication therapy. *Gut* **36**: 346–350

McPhail LC, Clayton CC and Snydermann R (1984) A potential second messenger role for unsaturated fatty acids: Activation of $Ca^{2+}$-dependent protein kinase. *Science* **224**: 622–625

Martelly I and Castagna M (1989) Protein kinase C and tumor promotors. *Curr Opin Cell Biol* **1**: 206–210

NIH Consensus Conference (1994) *Helicobacter pylori* in peptic ulcer disease. NIH consensus development panel. *JAMA* **272**: 65–69

Nishizuka Y (1986) Studies and perspectives of protein kinase C. *Science* **233**: 305–312

Ohkubo S, Yamada E, Endo T, Itoh H and Hidaka H (1984) Vitamin A acid-induced activation of $Ca^{2+}$-activated phospholipid-dependent protein kinase from rabbit retina. *Biochem. Biophys Res Commun* **118**: 460–466

Pfeiffer A, Rothbauer E, Wiebecke B, Pratschke E, Kramling HJ and Mann K (1990) Increased epidermal growth factor receptors in gastric carcinoma. *Gastroenterology* **98**: 961–967

Reynolds NJ, Talar HS, Baldassare JJ, Henderson PA, Elder JT, Voorhees JJ and Fisher GJ (1993) Differential induction of phosphatidylcholine hydrolysis, diacylglycerol formation and protein kinase C activation by epidermal growth factor and transforming growth factor-α in normal skin fibroblasts and keratinocytes. *Biochem J* **294**: 535–544

Richards GM (1974) Modifications of the diphenylamine reactions giving increased sensitivity and simplicity in the estimation of DNA. *Anal Biochem* **57**: 369–376

Roghani M, Da Silva C and Castagna M (1987) Tumor promotor chloroform is a potent protein kinase C activator. *Biochem Biophys Res Commun* **142**: 738–744

Rutten MJ, Dempsey PJ, Solomon TE and Coffey RJ (1993) Transforming growth factor α is a potent mitogen for primary cultures of guinea pig gastric mucous epithelial cells. *Am J Physiol* **265**: G361–G369

Tsujii M, Kawano S, Tsuji S, Nagano K, Ito T, Hayashi N, Fusamoto H, Kamado T and Tamura K (1992) Ammonia – a possible promotor in *Helicobacter pylori*-related gastric carcinogenesis. *Cancer Lett* **65**: 15–18

Wagner S, Beil W, Mai UEH, Bokemeyer C, Meyer HJ and Manns MP (1994) Interaction between *Helicobacter pylori* and human gastric epithelial cells in culture: effect of antiulcer drugs. *Pharmacology* **49**: 226–237

Wang L, Wilson EJ, Osburn J and Delvalle J (1996) Epidermal growth factor inhibits carbachol-stimulated canine parietal cell function via protein kinase C. *Gastroenterology* **110**: 469–477

Wightman PD and Raetz CRH (1984) The activation of protein kinase C by biologically active lipid moieties of lipopolysaccharide. *J Biol Chem* **259**: 10048–10052

© Cancer Research Campaign 1998